# Unusual presentation of aneurysmal bone cyst with scoliosis: a case report

**DOI:** 10.1186/s13256-022-03685-0

**Published:** 2022-11-28

**Authors:** Yousef M. Aljamaan, Hisham S. Alhathloul, Sami I. Aleissa, Majid S. Abaalkhail, Fahad H. Alhelal, Faisal M. Konbaz

**Affiliations:** 1 Department of Orthopedic Surgery, College of Medicine, King Fahad Hospital of the University, Imam Abdulrahman Bin Faisal University, Dammam, Saudi Arabia; 2 Department of Orthopedics, Armed Forces Hospital, King Abdulaziz Air Base, Dhahran, Saudi Arabia; 3 Department of Orthopedics, Ministry of The National Guard—Health Affairs, Riyadh, Saudi Arabia; 4 King Abdullah International Medical Research Center, Riyadh, Saudi Arabia; 5 King Saud Bin Abdulaziz University for Health Science, Riyadh, Saudi Arabia; 6 Spine Surgery Section, King Faisal Specialist Hospital and Research Center, Riyadh, Saudi Arabia

**Keywords:** Aneurysmal bone cyst, Scoliosis, Vertebral column resection

## Abstract

**Background:**

Aneurysmal bone cysts are benign bone tumors that not uncommonly involves the spine. However, this involvement can cause scoliosis, albeit rarely. This report focuses on the importance of proper management for complete tumor resection to prevent recurrence and spinal deformity.

**Case presentation:**

A 12-year-old Middle Eastern boy, with a history of T11 aneurysmal bone cyst resection and bone grafting carried out at another hospital, presented with spine deformity of 4 months’ duration. The deformity was not associated with pain or neurological deficit. A whole-spine magnetic resonance imaging with contrast confirmed the recurrence of the aneurysmal bone cyst. Posterior spinal instrumentation with corpectomy of T11 was then performed, and confirmed with histopathology the recurrence of aneurysmal bone cyst. Two years post-corpectomy, deformity correction was done from T5–L4.

**Conclusion:**

Management of aneurysmal bone cysts requires meticulous planning and full excision to prevent recurrence, especially in the growing spine. If neglected, it can cause major spinal deformities and cord compression, which places a medical burden on the patient and family. To avoid such complications, treating aneurysmal bone cysts along with scoliosis correction can prevent deformity progression.

## Background

Aneurysmal bone cysts (ABC) are benign aggressive bone lesions that account for 1.5–6% of primary bone tumors [1]. More than half of these tumors affect young patients under 20 years of age [2]. These tumors can occur in the spine in 6–20% of cases and specifically 34% in the thoracic spine [3]. Scoliosis and kyphosis manifest in roughly 10–15% of the cases [3].

Van Arsdale first described aneurysmal bone cysts as ossifying hematomas in 1893, and in 1942 the term ABC began to be used [4]. The pathophysiology of ABCs is debated, but most authors suggest that they develop owing to intercellular edema caused by the primary lesion expanding the surrounding loose stroma and permitting rupture of vessels into the microcysts under hemodynamic pressure, thereby causing a blood-filled cyst [5]. We present a case of a child who presented with symptoms of painless scoliosis with recurrence of the ABC. Subsequently, the patient underwent corpectomy and reconstruction with cage and bone graft. At follow-up 2 years post-surgery, the deformity had worsened and required long spinal fusion and deformity correction from T5–L4.

## Case presentation

A 12-year-old Middle Eastern boy, medically free, presented at our spine clinic complaining of spinal deformity for around 4 months. The deformity was not associated with pain or neurological symptoms. The patient provided a history of an aneurysmal bone cyst of T11 vertebra that had been resected with bone graft augmentation and posterior instrumentation T10–12 at a different hospital 2 years ago. The diagnosis of ABC was confirmed by histopathology. There was no family history of malignancy or scoliosis.

### Clinical findings

The examination was performed at the time of the initial presentation. It revealed mild scoliotic deformity upon Adam’s forward bend test with full spinal range of motion. There was no neurological deficit. Hips and feet were unremarkable.

**Fig. 1:**
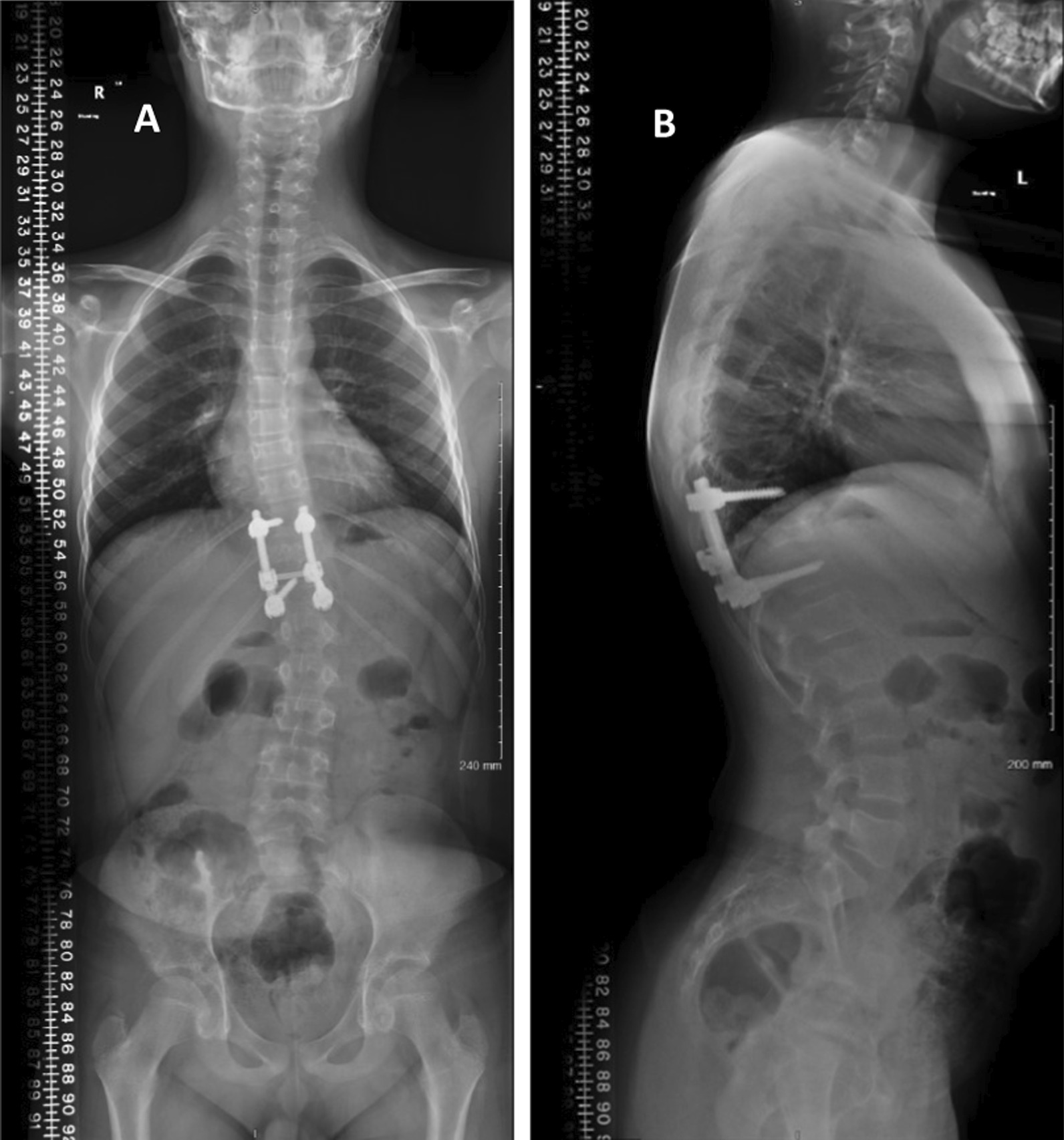
**A**, **B** Scoliosis X-ray **A** AP, and **B** lateral showing posterior spinal instrumentation from T10 to T12 with an open triradiate cartilage Reiser 0 along with left scoliotic deformity with Cobb angle of 24

**Fig. 2:**
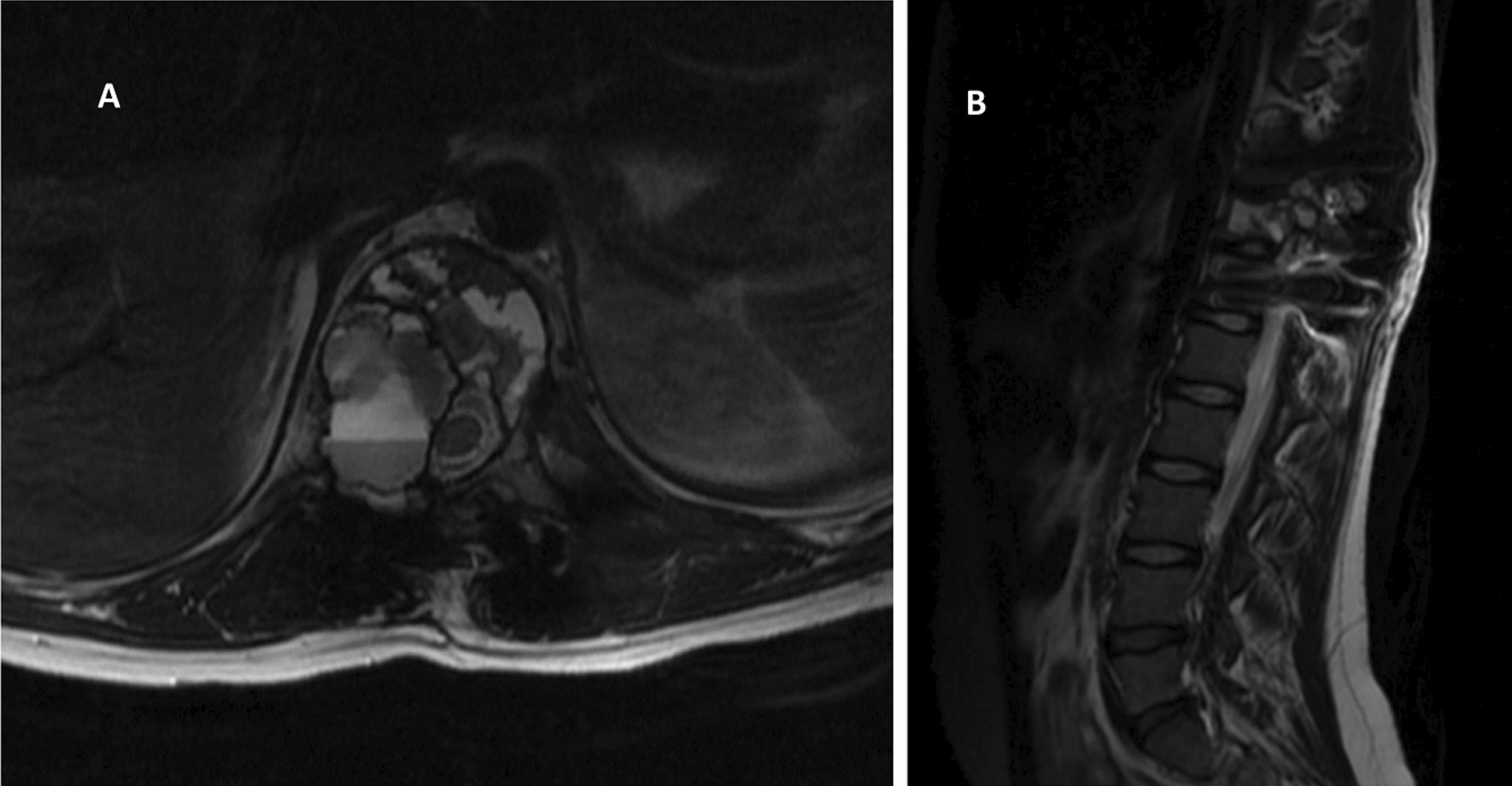
**A**, **B** MRI **A** axial and **B** sagittal showing Expansile bone lesion with fluid–fluid level at T11, keeping with ABC causing moderate compression fracture with extension into posterior elements causing sever right foraminal stenosis and mild cord compression

A full spine X-ray confirmed the finding of left scoliotic deformity with a Cobb angle of 24 along with posterior spinal instrumentation from T10–T12 with an open triradiate cartilage Rieser 0 (Fig. 1A, B). A full-spine magnetic resonance imaging (MRI) showed recurrence of the aneurysmal bone cyst (Fig. 2A, B). A diagnosis of juvenile idiopathic scoliosis was made.

### Therapeutic intervention

**Fig. 3:**
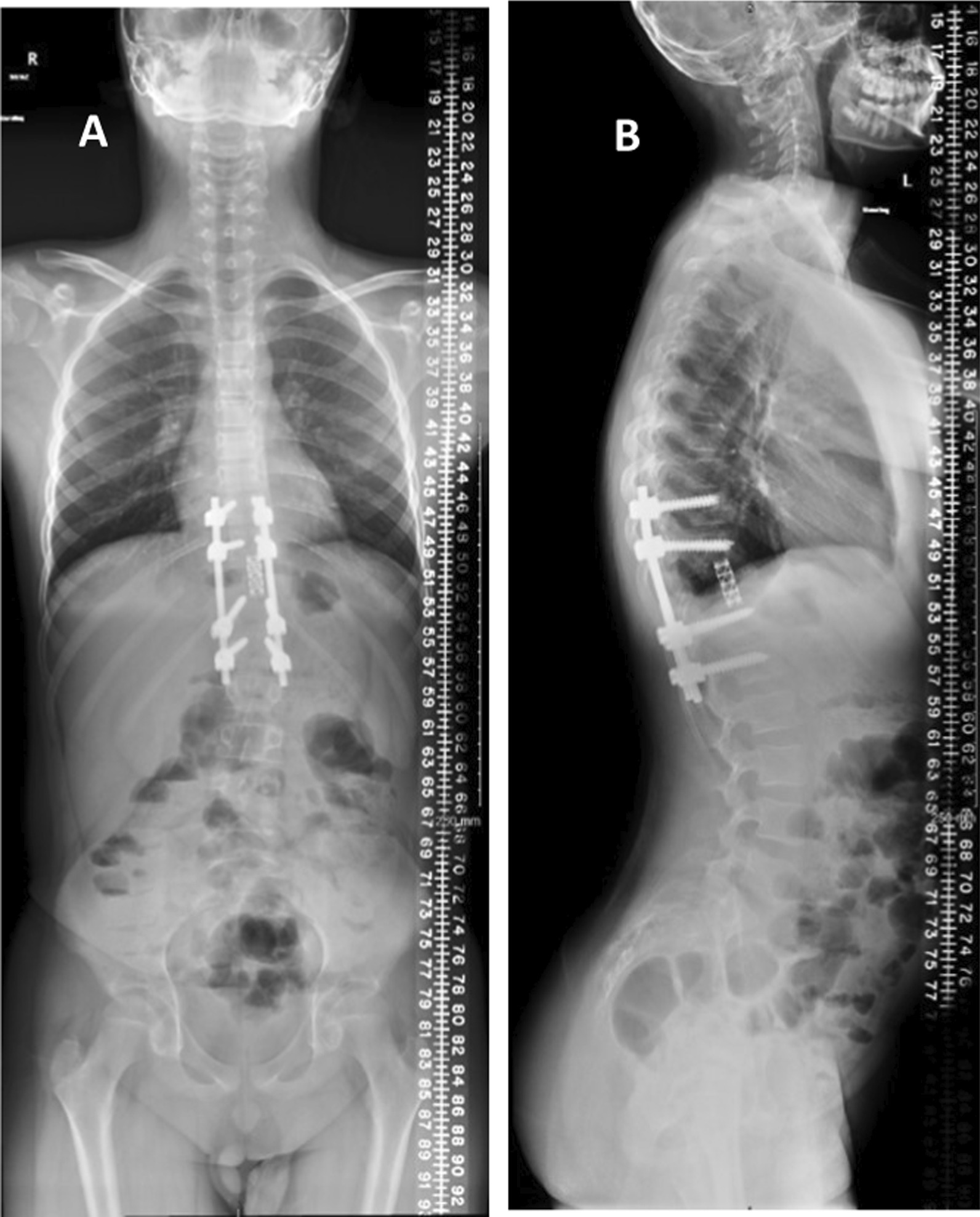
**A**, **B** scoliosis X-ray **A** anteroposterior (AP) and **B** lateral, 4 months post first surgery showing T11 resected vertebral body with cage placement, thoracolumbar fixation rods and screws, with a scoliosis measure of 6.5°

**Fig. 4:**
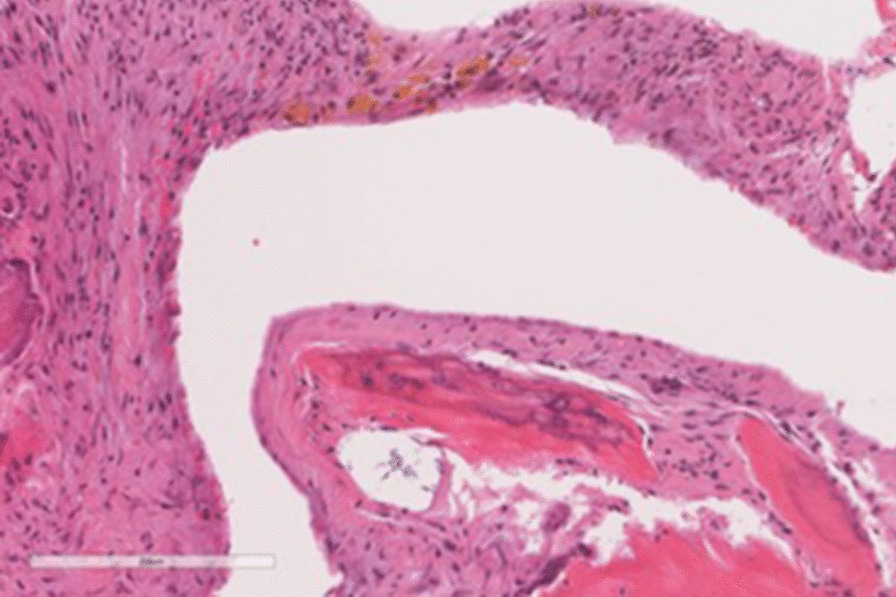
Photomicrograph (haematoxylin–eosin-stained × 20) of the tumor showing fragments of bone tissue cystic space walled by fibroblastic tissue, area of hemorrhage, multinucleated giant cells, fibrosis, and hemosiderin deposition

**Fig. 5:**
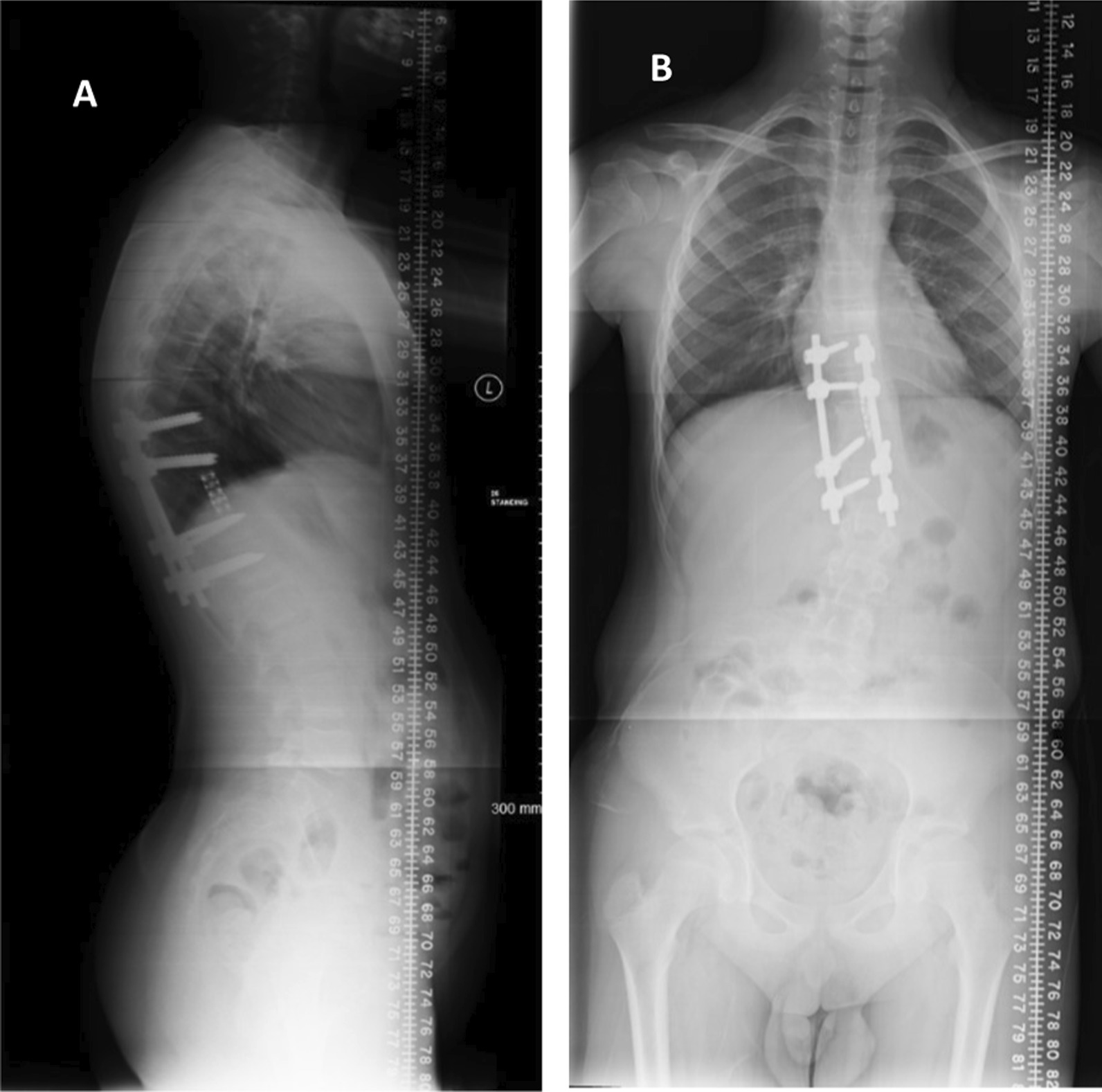
**A**, **B** Scoliosis X-ray **A** AP and **B** lateral, 1 year post first surgery showing T11 resected vertebral body with cage placement thoracolumbar fixation rods and screws with S-shape scoliosis Cobb angle of 11°

**Fig. 6:**
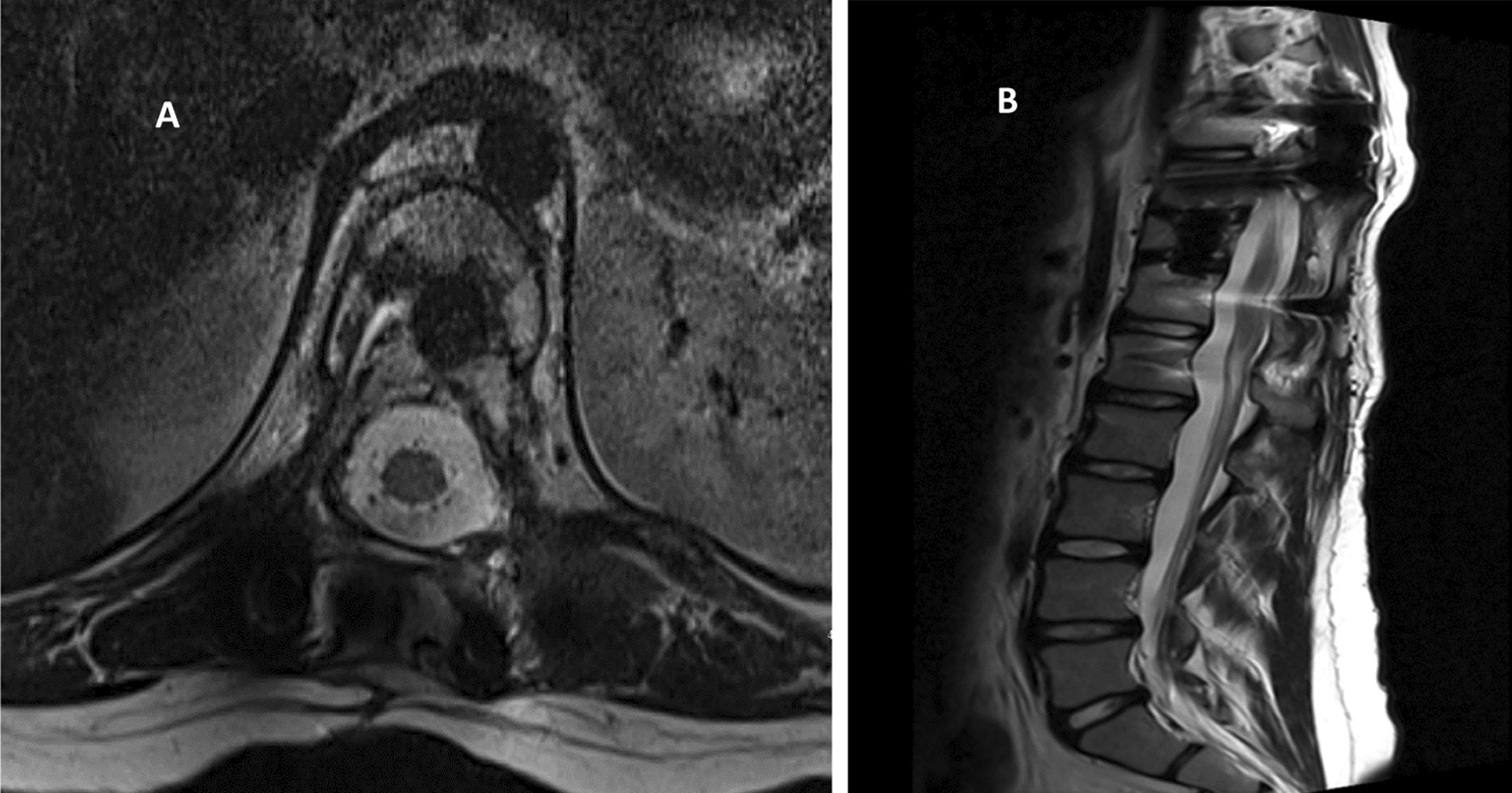
**A**, **B** MRI **A** axial and **B** sagittal showing partially resected T11 with presence of a vertical mesh cage in place with no evidence of residual or recurrent aneurysmal bone cysts

**Fig. 7:**
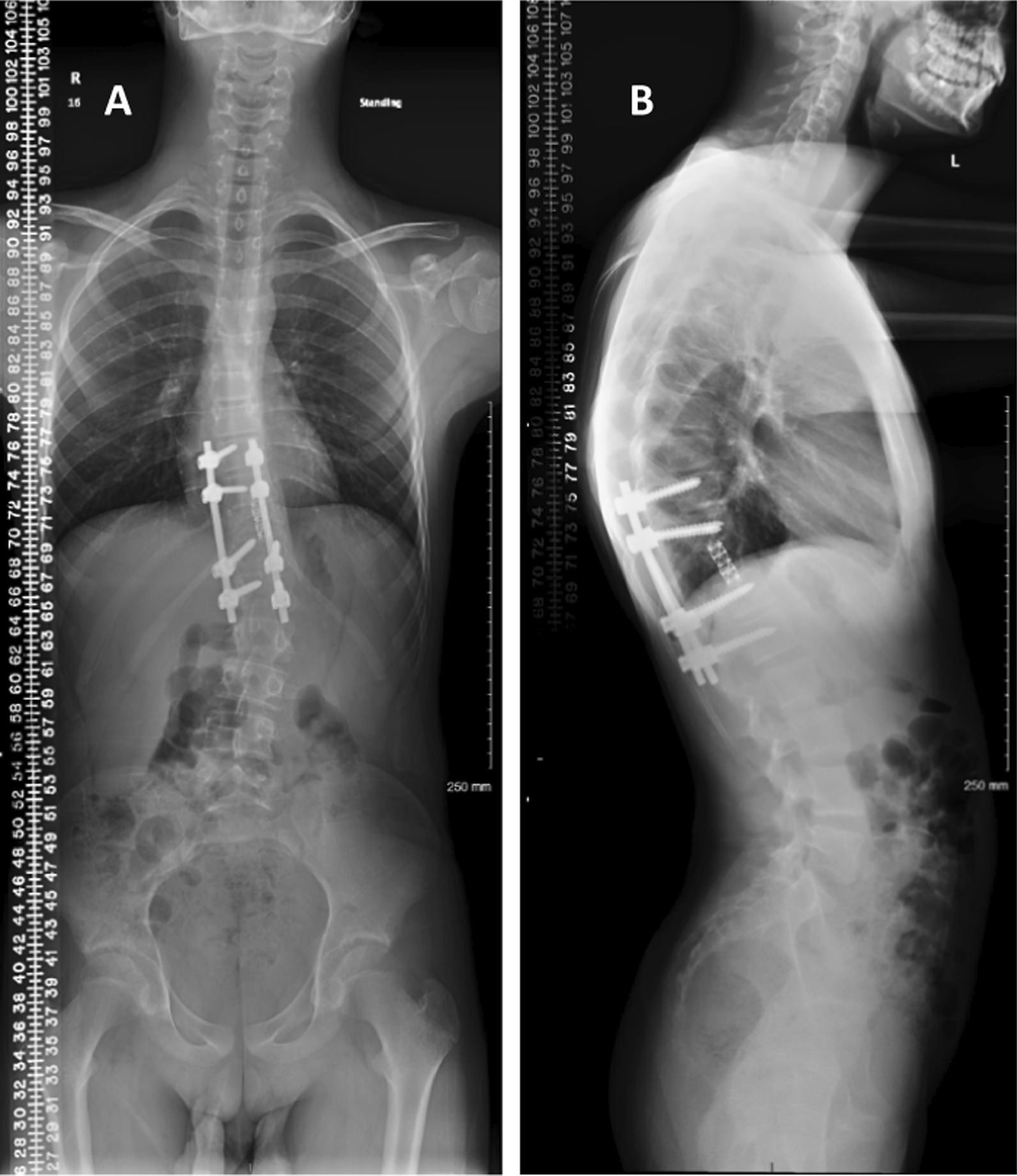
**A**, **B** Scoliosis X-ray **A** AP and **B** lateral, 2 years post first surgery showing T11 resected vertebral body with cage placement thoracolumbar fixation rods and screws with S-shape scoliosis with Cobb angle of 30°

**Fig. 8:**
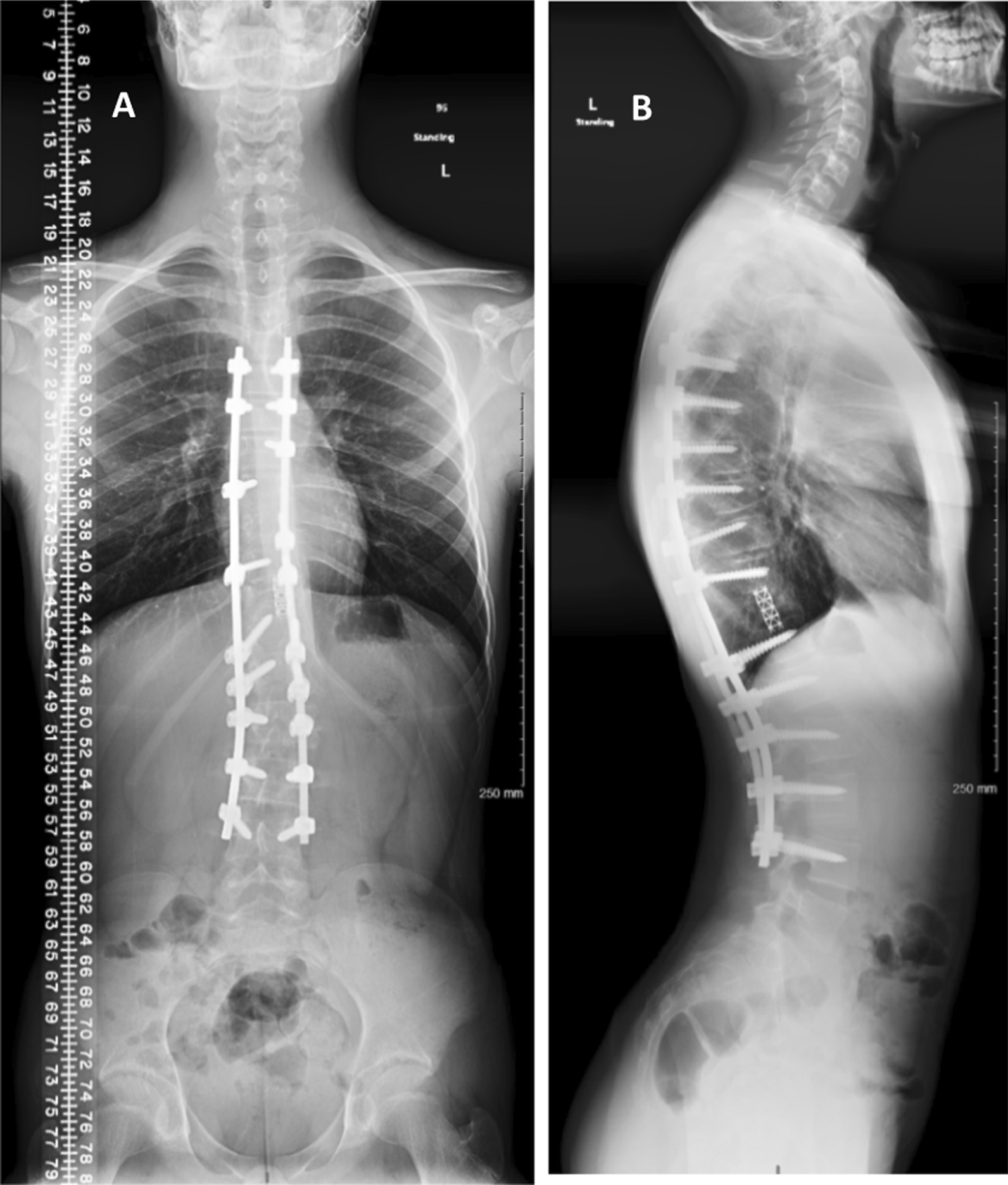
**A**, **B** Scoliosis X-ray **A** AP and **B** lateral, 1 year post revision surgery showing T11 resected vertebral body with cage placement, and posterior spinal fixation from T5 to L4 with scoliosis improvement and no complication is seen

Six weeks after presentation, the patient was admitted for surgery. Prior to surgery the patient was taken for embolization, but following an angiogram, no embolization was performed in view of the mild vascularity and the origin of posterior spinal artery from the same level. The procedure consisted of revision posterior spinal fusion and instrumentation, T9-L1 (5 levels), laminectomy, T10-T12 (2 levels), open biopsy T11, tumor resection, navigation-assisted T11 revision of corpectomy, and anterior fusion T11 with cage (Fig. 3A, B). The final pathology result confirmed the recurrence of ABC (Fig. 4). Upon follow-up, we noticed that the child had developed juvenile idiopathic thoracolumbar scoliosis, which increased to 11 degrees (Fig. 5A, B). One year post-surgery, an MRI confirmed no recurrence of ABC (Fig. 6A, B). Two years post-surgery, scoliosis had reached 30 degrees (Fig. 7A, B). Regarding growth stage, the child was at Risser stage 0. A trial of brace treatment was carried out, but unfortunately the child was not compliant with the brace. An MRI done at that time showed no recurrence. Hence the decision was made to proceed with scoliosis correction and extend the fusion to T5 proximally and to L4 distally. At 1-year follow-up, scoliosis x-ray (Fig. 8A, B) showed maintenance of scoliosis correction without implant complications, and a final MRI was done which showed no recurrence. The patient believed that the treatment had improved his quality of life and increased his satisfaction.

## Discussion

We present a case of an aggressive aneurysmal bone cyst of the thoracic spine that presented with juvenile scoliosis and showed recurrence of the ABC; MRI confirmed the diagnosis, along with a histopathological examination. The patient underwent posterior spinal fusion and instrumentation of five levels from T9 to L1 via a one-stage posterior and anterior approach, decompression, open biopsy, complete tumor resection, and anterior fusion of T11. Post resection of the tumor, the initial scoliosis was resolved. Upon follow-up, there was no recurrence of the tumor; however, there was progression of scoliosis. The patient was treated with one-stage long spinal fusion and deformity correction from T5 to L4 via an anterior and posterior approach. To our knowledge, this is the first case in literature that reports progressive spinal deformity along with complete tumor resection that was treated with a one-stage anterior and posterior approach. One of the most important factors to prevent tumor recurrence is the extent of tumor resection. Zenonos et al. reported that of 14 patients, two showed recurrence owing to incomplete tumor resection [6]. Recurrence of ABC is around 10–44%, and 90% recur within 2 years [7]. Rahimizadeh et al. published a case in 2013 of ABC at C7 level, where the patient underwent a one-stage anterior and posterior approach and showed no recurrence after 1 year [8].

ABC usually presents in the second decade of life. It accounts for approximately 1.5–6% of all primary bone tumors, and 15–20% of all primary spine tumors [1][1]. The areas of predilection are the femur, tibia, humerus, spine, pelvis, ribs, and small bones of the hands and feet [10]. Most commonly, the posterior elements of the spine are involved [11]. Some studies report more incidence in the lumbar spine [12, 13]. Others report more incidence in the cervical and thoracic spine [14, 15], or equal incidence in the thoracic and lumbar regions [6, 16]. Patients present with neurological symptoms in 60–70% of cases [17]. Many neurological signs and symptoms may be noted, ranging from backache and radiculopathy to paraplegia depending on the level. Acute spinal cord compression may occur if there is a break in the posterior cortex of the body [18]. Scoliosis and kyphosis are noted in 10–15% of cases owing to back pain, spasms, or weakness [3]. Konbaz et al. mentioned that coexistence of tumor with scoliosis is present in the literature [19]. Many techniques should be used to arrive at the diagnosis and for preoperative planning. X-ray shows excentric, osteolytic, expansive, and trabeculated lesions with fine-walled cystic cavities. Loss of cortical contours can mimic a malignant lesion [20]. A spine X-ray should be performed as an initial workup examination, although it does not reveal the presence of ABC [21]. CT scans also show internal septation and eggshell appearance with calcified rim, as well as fluid level [22], but these findings were not exclusive. For fluid, the patient should stay 10 minutes in position to obtain enough separation of the materials of different attenuation. CT was also needed for preoperative fusion and instrumentation planning of pedicles and transverse processes [23]. MRI allows for visualizing a well-defined lesion with lobulated contours and liquid inside it. Internal septa showed a decrease in the signal, presumably owing to the presence of fibrous tissue. Liquid characteristics of ABC are better explored in MRI [24]. Kransdorf et al. used MRI even for follow-up as it is superior to biplanar radiographs [7]. Bone scintigraphy showed increased uptake of radionuclides in the peripheral area of the lesion in most cases [21]. In some patients, the radiological findings are inconclusive, so an open biopsy is necessary to establish the definitive diagnosis because it can determine grading and signs of tumor malignancy [25]. The character of ABC histology is cavernous channels surrounded by a spindle cell stroma with osteoclast-like giant cells and osteoid production [26]. Differential diagnoses are simple bone cysts, hyperparathyroidism brown tumor, giant cell tumor, chondrosarcoma, osteosarcoma, and Ewing’s sarcoma [25]. At the microscopic level, findings are dense and cellular composition of the cyst, containing plump stromal cells, multinuclear giant cells, and thin-walled blood vessels, or are in the form of preponderantly fibrous tissue with enlarging vascular spaces [27].

Treatment of ABC is also controversial. Because of their unique anatomical structure and function, there are special considerations when managing ABCs of the spine. Treatment options are curettage with or without bone grafting, complete excision, arterial embolization, intralesional drug injections, and radiation [22]. Curettage alone has shown highly variable recurrence rates, reaching 59% in some studies [28]. Vertebroplasty or kyphoplasty can be used after curettage to reinforce bone defects [29]. Radiotherapy is recommended in inoperable cases [14]. Arterial embolization can be used as an adjunct to surgery, but it has also been employed as a primary treatment in ABC lesions that are difficult to access when surgery is not feasible [18]. According to Park et al., treatment of ABC is usually surgical, but embolization and radiotherapy can be used without surgery when it will cause significant morbidity plus medical management with denosumab [30]. Regarding our patient, his initial surgery at a different hospital was not done appropriately. Therefore, he presented with recurrence of tumor and scoliosis. The second surgery, which we carried out, achieved complete tumor resection and stabilization; however, we did not address his scoliosis. We had to carry out a third procedure to address his scoliosis with deformity correction and fusion. What is unique about our case is that post-tumor resection, the patient continued to grow and his scoliosis progressed; however, there was no tumor recurrence. This young patient had to undergo three spine surgeries in the space of almost 4 years. To achieve complete tumor resection and prevent recurrence, careful planning in the index surgery with meticulous tumor resection along with spinal reconstruction and fusion is extremely important and might have spared the patient all these sequelae. The literature lacks a well-structured protocol in such cases as to whether the tumor alone is treated or both the tumor and spinal deformity are treated at the same time, especially for a patient who is still growing. However, in young patients with a growing spine it is important to preserve the alignment of the spine and allow natural development until growth is completed [31]. We believe more cases need to be published to establish a clear guideline for proper management of such cases and to avoid future complications.

## Conclusion

Recurrent ABC can be challenging to manage as it can result in spinal deformity, instability, and surgical impairment, posing a significant burden on the child and their family. Although in cases of tumor coexisting with scoliosis the usual recommendation is to manage the tumor first, we believe that discussing the option of managing both problems at one stage is a valid consideration. Therefore, identification of an appropriate index treatment that decreases recurrence rates and complications is crucial for the treatment of ABC.

## Data Availability

All data generated or analyzed during this study are included in this published article and its supplementary information files.

## Abbreviations

ABC: Aneurysmal bone cyst
MRI: Magnetic resonance imaging

## References

[1] Burch S, Hu S, Berven S. Aneurysmal bone cysts of the spine. Neurosurg Clin N Am. 2008;41:1–7. 10.1016/j.nec.2007.09.005 18156046

[2] Daszkiewicz P, Roszkowski M, Grajkowska W. Aneurysmal bone cyst of skull and vertebrae in children. Analysis of own material and review of the literature. Folia Neuropathol. 2004;42:25–30. 15119742

[3] Codd PJ, Riesenburger RI, Klimo P, Slotkin JR, Smith ER. Vertebra plana due to an aneurysmal bone cyst of the lumbar spine case report and review of the literature. J Neurosurg. 2006;105:490–5. 10.3171/ped.2006.105.6.490 17184084

[4] Sartawi M, Quateen A, Nataraj A, Medairos R. Spinal intradural aneurysmal bone cyst: a case report. World Neurosurg. 2015;84(2):593.e1-5. 10.1016/j.wneu.2015.02.012 25769483

[5] Rossi G, Rimondi E, Bartalena T, *et al*. Selective arterial embolization of 36 aneurysmal bone cysts of the skeleton with N-2-butyl cyanoacrylate. Skeletal Radiol. 2010;39(2):161–7. 10.1007/s00256-009-0757-z 19669138

[6] Zenonos G, Jamil O, Governale LS, Jernigan S, Hedequist D, Proctor MR. Surgical treatment for primary spinal aneurysmal bone cysts: experience from Children’s Hospital Boston. J Neurosurg Pediatr. 2012;9(3):305–15. 10.3171/2011.12.PEDS11253 22380960

[7] Deventer N, Schulze M, Gosheger G, de Vaal M, Deventer N. Primary aneurysmal bone cyst and its recent treatment options: a comparative review of 74 cases. Cancers. 2021;13:2362. 10.3390/cancers13102362 PMC8153560 34068844

[8] Rahimizadeh A, Taghinedjadi O, Rahimizadeh A. One stage management of aggressive cervicothoracic aneurysmal bone cyst in a 14-year old girl: report of a case and review of the literature. World Spinal Column J. 2013;4:109–19.

[9] Cottalorda J, Kohler R, Sales de Gauzy J, *et al*. Epidemiology of aneurysmal bone cyst in children: a multicenter study and literature review. J Pediatr Orthop B. 2004;13(6):389–94. 10.1097/01202412-200411000-00008 15599231

[10] Jaiswal A, Vijay V, Kori P, Shukla R. Aneurysmal bone cyst of thoracic spine: case report and brief review of literature. BMJ Case Rep. 2013. 10.1136/bcr-2013-009265. PMC3702870 23814197

[11] Hay MC, Paterson D, Taylor TK. Aneurysmal bone cysts of the spine. J Bone Jt Surg Br. 1978;60B(3):406–11. 10.1302/0301-620X.60B3.681419 681419

[12] Capanna R, Albisinni U, Picci P, Calderoni P, Campanacci M, Springfield DS. Aneurysmal bone cyst of the spine. J Bone Jt Surg Am. 1985;67(4):527–31. 3980496

[13] Boriani S, De Iure F, Campanacci L, *et al*. Aneurysmal bone cyst of the mobile spine: report on 41 cases. Spine (Phila Pa 1976). 2001;26(1):27–35. 10.1097/00007632-200101010-00007 11148642

[14] Vergel De Dios AM, Bond JR, Shives TC, McLeod RA, Unni KK. Aneurysmal bone cyst. A clinicopathologic study of 238 cases. Cancer. 1992;69(12):2921–31. 10.1002/1097-0142(19920615)69:12<2921::aid-cncr2820691210>3.0.co;2-e 1591685

[15] Papagelopoulos PJ, Currier BL, Shaughnessy WJ, *et al*. Aneurysmal bone cyst of the spine. Management and outcome. Spine (Phila Pa 1976). 1998;23(5):621–8. 10.1097/00007632-199803010-00018 9530795

[16] Garg S, Mehta S, Dormans JP. Modern surgical treatment of primary aneurysmal bone cyst of the spine in children and adolescents. JPediatr Orthop. 2005;25(3):387–92. 10.1097/01.bpo.0000152910.16045.ee 15832161

[17] Liu JK, Brockmeyer DL, Dailey AT, Schmidt MH. Surgical management of aneurysmal bone cysts of the spine. Neurosurg Focus. 2003;15:E4. 15323461

[18] Chen SH, Huang TJ, Hsueh S, Lee YY, Hsu RW. Unusual bleeding of aneurysmal bone cyst in the upper thoracic spine. Chang Gung Med J. 2002;25:183–9. 12022739

[19] Konbaz FS, Althunayan TA, Alzahrani MT, Altawayjri IA. Aggressive L3 vertebral hemangioma coexisting with adult thoracolumbar scoliosis: case report. N Am Spine Soc J. 2020;8(5): 100040. 10.1016/j.xnsj.2020.100040 PMC8819962 35141607

[20] Lange T, Stehling C, Fröhlich B, Klingenhöfer M, Kunkel P, *et al*. Denosumab: a potential new and innovative treatment option for aneurysmal bone cysts. Eur Spine J. 2013;22:1417–22. 10.1007/s00586-013-2715-7 PMC3676549 23455951

[21] Zileli M, Isik HS, Ogut FE, Is M, Cagli S, Calli C. Aneurysmal bone cysts of the spine. Eur Spine J. 2013;22(3):593–601. 10.1007/s00586-012-2510-x PMC3585636 23053752

[22] Tsai JC, Dalinka MK, Fallon MD, Zlatkin MB, Kressel HY. Fluid-fluid level: a nonspecific finding in tumors of bone and soft tissue. Radiology. 1990;175:779–82. 10.1148/radiology.175.3.2160676 2160676

[23] Chan MS, Wong YC, Yuen MK, Lam D. Spinal aneurysmal bone cyst causing acute cord compression without vertebral collapse: CT and MRI findings. Pediatr Radiol. 2002;32:601–4. 10.1007/s00247-001-0648-5 12136354

[24] Kransdorf MJ, Sweet DE. Aneurysmal bone cyst: concept, controversy, clinical presentation, and imaging. AJR Am J Roentgenol. 1995;164(3):573–80. 10.2214/ajr.164.3.7863874 7863874

[25] Burch S, Hu S, Berven S. Aneurysmal bone cysts of the spine. Neurosurg Clin N Am. 2008;19(1):41–7. 10.1016/j.nec.2007.09.005 18156046

[26] Al-Shamy G, Relyea K, Adesina A, Whitehead WE, Curry DJ, Luerssen TG, Jea A. Solid variant of aneurysmal bone cyst of the thoracic spine: a case report. J Med Case Rep. 2011;5(261):1–6. 10.1186/1752-1947-5-261 PMC3141711 21718507

[27] O’Brien J, Ward E, Doody O, Ryan M. A case of back pain associated with neurology in a young man. Ir J Med Sci. 2009;178:373–5. 10.1007/s11845-008-0170-y 18516661

[28] Biesecker JL, Marcove RC, Huvos AG, Miké V. Aneurysmal bone cysts. A clinicopathologic study of 66 cases. Cancer. 1970;26(3):615–25. 10.1002/1097-0142(197009)26:3<615::aid-cncr2820260319>3.0.co;2-i 5458269

[29] Ropper AE, Cahil KS, Hanna JW, McCarthy EF, Gökaslan ZL, Chi JH. Primary vertebral tumors: a review of epidemiologic, histological and imaging findings, part 1: benign tumors. Neurosurgery. 2011;69:1171–80. 10.1227/NEU.0b013e31822b8107 21725252

[30] Park HY, Yang SK, Sheppard WL, Hegde V. Current management of aneurysmal bone cysts. Curr Rev Musculoskelet Med. 2016;9:435–44. 10.1007/s12178-016-9371-6 PMC5127951 27778155

[31] Lambot-Juhan K, Pannier S, Grévent D, Péjin Z, Breton S, Berteloot L, Emond-Gonsard S, Boddaert N, Glorion C, Brunelle F. Primary aneurysmal bone cysts in children: percutaneous sclerotherapy with absolute alcohol and proposal of a vascular classification. Pediatr Radiol. 2012;42(5):599–605. 10.1007/s00247-011-2312-z 22237479

